# A review of decomposition methods for brain states estimation

**DOI:** 10.1186/s12938-026-01542-5

**Published:** 2026-02-18

**Authors:** Guoqiang Hu, Jinxing Wang, Ziyi Shui, Tianyang Wang, Deqing Wang, Siwen Luo, Hongbo Liu, Xinqiang Xie, Lisa D. Nickerson

**Affiliations:** 1 College of Artificial Intelligence, Dalian Maritime University, Dalian, China; 2 Huzhou Key Laboratory of Brain Science and Child Learning, Huzhou, China; 3 State Key Laboratory of Robotics, Shenyang Institute of Automation, Chinese Academy of Sciences, Shenyang, China; 4 Key Laboratory of Marine Robotics, Shenyang, Liaoning China; 5 Affiliated Zhongshan Hospital of Dalian University, Dalian, China; 6 Imaging Center, McLean Hospital, Belmont, MA USA; 7 Department of Psychiatry, Harvard Medical School, Boston, MA USA

**Keywords:** fMRI, Brain state, Matrix decomposition, Tensor decomposition

## Abstract

In the rapidly advancing field of neuroscience, sophisticated imaging techniques such as functional magnetic resonance imaging (fMRI) enable detailed analysis of brain activity. Researchers increasingly seek to disentangle distinct brain states, recognizing that fMRI data typically comprise a mixture of these states. To enable independent analysis of individual brain states, numerous methodologies have been proposed, each requiring careful consideration in practical application. This review provides a comprehensive survey of decomposition methods, covering classical, probabilistic, and tensor-based approaches and their applications. Furthermore, the review discusses additional methodological considerations essential for the effective use of these techniques. By comparing decomposition algorithms with other widely used techniques in fMRI data analysis, this review highlights their methodological strengths and limitations, and further demonstrates their broad applicability for extracting brain states from fMRI data.

**Supplementary Information:**

The online version contains supplementary material available at 10.1186/s12938-026-01542-5.

## Introduction

Ever since Richard Caton performed the first recordings from the mammalian brain in the late 1800s, it has been recognized that patterns of brain activity are dynamically dependent on the behavioral state of the animal. Today, with advances in functional magnetic resonance imaging (fMRI) and electrophysiological techniques, it is possible to monitor dynamic brain activity across large portions of the brain at a wide range of spatial and temporal scales. These technological advances have provided new insights into the organization and dynamics of the waking brain. These findings indicate that activity in the awake human brain varies continuously, transitioning between distinct and organized brain states [1].

Transitions between different brain states are associated with substantial changes in global brain activity. In clinical applications, understanding brain states can inform the treatment of neurological and psychiatric disorders, such as epilepsy and depression [2]. In the field of cognitive enhancement, researchers investigate technologies aimed at enhancing cognitive abilities by studying brain states, for example through neurofeedback [3] or brain stimulation. From a mental health perspective, understanding the neural basis of different emotions and cognitive states [4] could improve therapeutic interventions and support the development of more effective diagnostic tools and treatment strategies. From a neuroscientific perspective, the study of brain states bridges multiple disciplines within neuroscience and psychology, providing a holistic view of brain function as an integrated system. This line of research also emphasizes the dynamic nature of the brain, particularly its transitions between different states in response to internal and external stimuli [5]. Overall, in-depth research on brain states not only expands our understanding of the relationships between the brain, behavior, and health, but also provides a strong theoretical and practical foundation for interdisciplinary research.

A wide range of statistical techniques have been developed to investigate brain states and their dynamics. However, the fMRI signal—specifically, the blood oxygenation level-dependent (BOLD) response—is an indirect and noisy proxy for neural activity. It is subject to various sources of confounds, including head motion, physiological noise, and neurovascular coupling, making reliable estimation of brain states challenging. To address these limitations, researchers have proposed diverse modeling strategies. Model-driven approaches, such as Dynamic Causal Modeling (DCM) [6], Hidden Markov Models (HMM) [7], and Co-Activation Pattern analysis (CAP) [8], aim to infer latent brain states based on predefined assumptions about neural dynamics. In contrast, data-driven decomposition methods have gained increasing attention for their ability to extract latent brain states from corrupted signals without requiring explicit dynamic models.

Within this data-driven paradigm, existing decomposition methods differ substantially in their underlying mathematical assumptions and data representations. Matrix-based approaches, including spatial independent component analysis (ICA) [9, 10], temporal ICA [11], principal component analysis (PCA) [12], and non-negative matrix factorization (NMF) [13], vary in how they model spatial and temporal dependencies. More recently, tensor-based methods—such as Tensor Component Analysis (TCA) [14], Tensor ICA [15], and Sparsity Nonnegative Tensor Decomposition (SNTD) [16]—have been proposed to exploit the intrinsic multi-way structure of fMRI data across time, space, subjects, or experimental conditions. Despite their widespread use, these approaches are often developed and applied in isolation, leading to a fragmented methodological landscape. The lack of a unified perspective makes it difficult to compare methods systematically or to select appropriate techniques for specific brain state analysis tasks. This fragmentation highlights the need for a coherent framework that can integrate different decomposition strategies and clarify their relationships, strengths, and limitations.

In this review, we systematically examine nine representative data decomposition methods for brain state estimation from fMRI signals. Our analysis emphasizes their underlying mathematical assumptions, implementation strategies, and practical challenges encountered in real-world applications. In addition, we critically discuss how these decomposition approaches relate to—and fundamentally differ from—other widely used methods for dynamic brain state analysis.

## Methods review

**Fig. 1:**
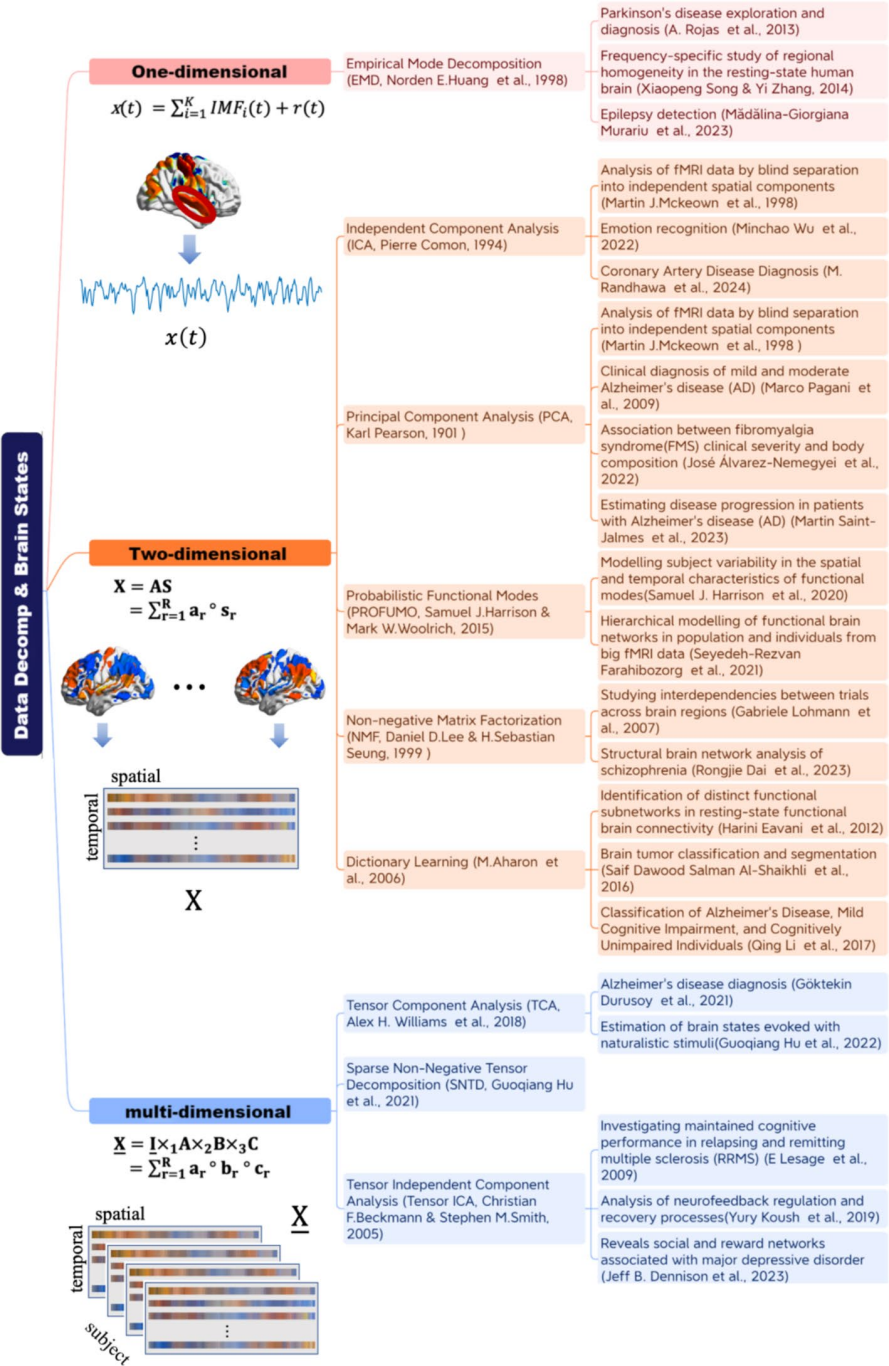
Illustration of data decomposition methods applied on brain state estimation and its clinical applications. To provide a structured overview, the methods are organized by the dimensionality of the input data: one-dimensional approaches operating on temporal signals; two-dimensional matrix decompositions applied to space × time; and multi-dimensional tensor decompositions that preserve the native multi-mode structure of fMRI data. Representative methods and their typical neuroimaging applications are shown for each category. The symbol $^\circ$ represents outer product and ${\times }_{{\boldsymbol{i}}}$ represents the product of a tensor's *i*^th^ dimension with a matrix

As illustrated in Fig. 1, we categorize data decomposition methods into one-way, two-way, and multi-way approaches. We then introduce the mathematical frameworks underlying each technique for the decomposition of fMRI data represented in matrix or tensor form. For all methods discussed in this review, we assume that the fMRI data have undergone standard preprocessing, readers are referred to [17, 18] for representative preprocessing pipelines.

Data decomposition typically begins by reorganizing the 4D fMRI data of each subject into a two-dimensional matrix, with time points (scans) along one dimension and spatial units (voxels or regions of interest) along the other. Multi-subject data can then be combined in different ways: temporal concatenation yields a two-dimensional matrix of size $\left({N}_{subjects}\times {N}_{time points}\right)\times {N}_{voxels}$, whereas stacking individual subject matrices along a third dimension results in a three-way tensor with dimensions time × space × subject. In addition, time–frequency analysis can be performed to introduce an explicit frequency mode for frequency-domain investigations. Data decomposition methods are subsequently applied to these matrix or tensor representations to identify latent brain states.

## One-dimensional approaches

### Empirical mode decomposition

Empirical mode decomposition (EMD) is an adaptive time–frequency signal processing method proposed by Huang et al. for the analysis of nonlinear and non-stationary signals [19]. Originally developed for seismic signal analysis, EMD has since been widely applied in diverse fields, including biomedical engineering, mechanical fault diagnosis, and financial analysis. In neuroscience, Song et al. first applied EMD to fMRI data, decomposing the signals into intrinsic mode functions (IMFs) that represent distinct characteristic time scales within the data [20]. Each IMF corresponds to a narrow-band oscillatory mode with relatively stable amplitude and frequency over time. The extraction of an IMF is governed by two conditions: (1) the numbers of extrema and zero crossings must be equal or differ by at most one over the entire signal, and (2) the local mean at any time point—defined as the average of the upper and lower envelopes formed by local maxima and minima—must be zero.

**Fig. 2:**
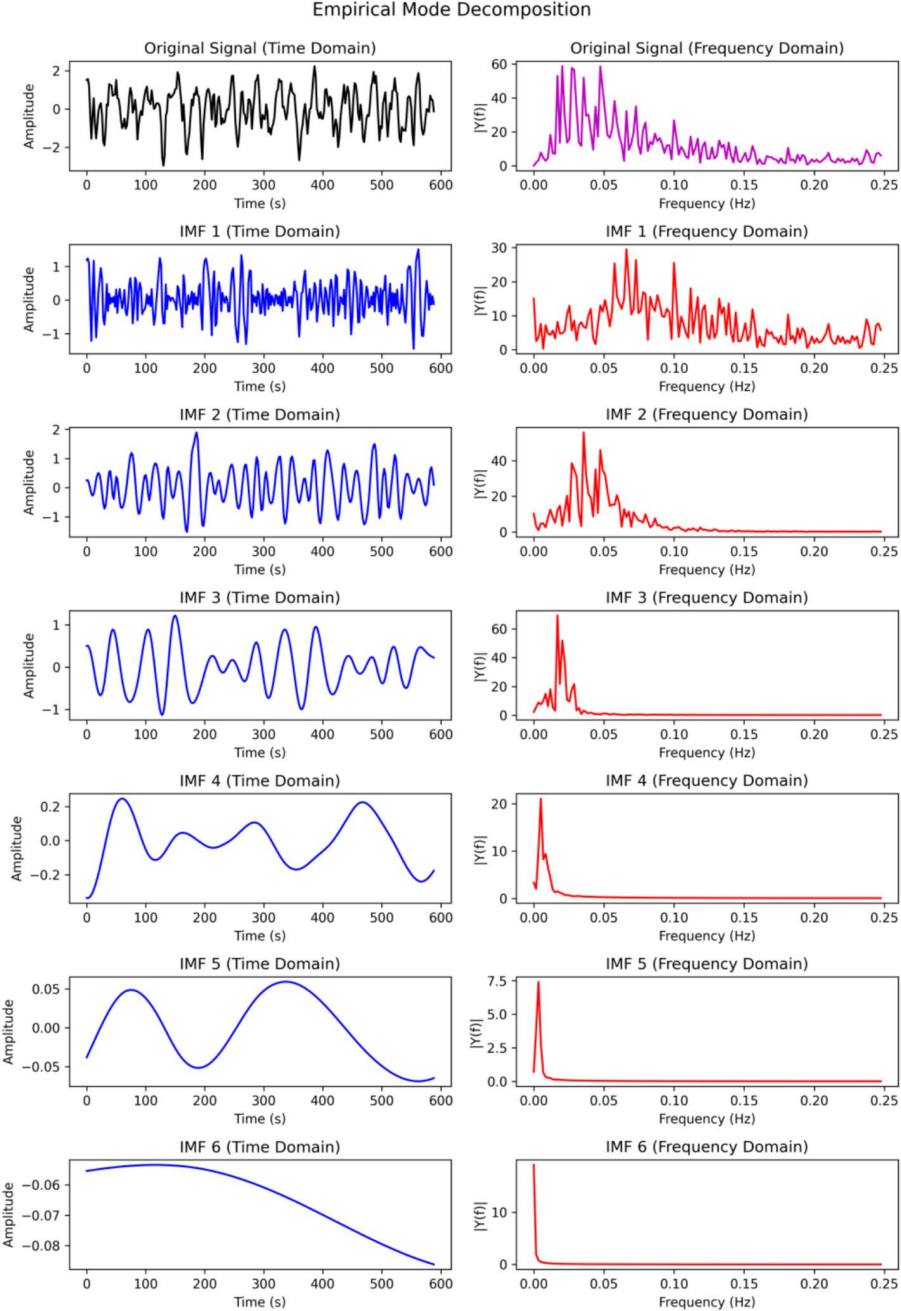
Illustration of Empirical Mode Decomposition. The top row shows the original signal (left) and its Fourier spectrum (right). Rows 2–7 display the decomposed intrinsic mode functions (IMF1–IMF6), with each IMF shown alongside its corresponding frequency-domain representation

As shown in Fig. 2, EMD is performed recursively, starting from the original signal and progressively extracting IMFs through local extrema identification, envelope construction, and mean removal. The IMF obtained at each iteration captures the most rapidly varying (i.e., highest-frequency) component of the data. After extraction, this component is subtracted from the signal, and the same procedure is applied to the residual until no further IMFs can be extracted. Detailed algorithmic descriptions of EMD, together with information on readily available software implementations, are provided in the Appendix.

Derivative methods of EMD have played an important role in signal processing and data analysis, including ensemble empirical mode decomposition (EEMD) [21] and multivariate empirical mode decomposition (MEMD) [22]. EEMD was proposed as an improvement over traditional EMD to alleviate mode mixing and spurious artifacts by introducing a noise-assisted decomposition strategy. By averaging results across multiple noise realizations, EEMD improves the robustness and reliability of the extracted intrinsic mode functions (IMFs), making it particularly suitable for noisy or nonstationary signals. However, the introduction of noise and repeated decompositions increases the computational cost, rendering EEMD less suitable for real-time or large-scale applications. MEMD extends EMD to multivariate signals and enables the simultaneous decomposition of multiple channels while preserving inter-channel relationships. This property makes MEMD especially appropriate for multichannel neuroimaging data, such as fMRI or MEG, where cross-channel alignment and scale consistency are critical. In contrast, for single-channel signals, MEMD may be unnecessary or inefficient and can become computationally demanding as the number of channels increases. Other EMD variants, including local EMD, fast EMD, and time–frequency EMD, have been developed to address specific practical constraints such as computational efficiency or enhanced time–frequency resolution. While these variants provide flexible tools for signal analysis and feature extraction, their applicability is often problem-dependent, and overly specialized variants may sacrifice decomposition interpretability or stability. Therefore, the selection of EMD variants should be guided by signal dimensionality, noise characteristics, and computational requirements.

Although EMD provides a flexible and data-driven framework for decomposing nonstationary fMRI time series, several methodological challenges must be acknowledged. First, EMD is known to suffer from edge artifacts, particularly when the signal length is short or when spline interpolation at the boundaries is poorly constrained. These artifacts may distort instantaneous frequency estimation and introduce artificial oscillatory patterns near the beginning and end of the time series. Second, mode mixing—where oscillations of similar scales are split across multiple IMFs or oscillations of different scales appear in the same IMF—remains a common issue, especially in fMRI where noise levels are high and component separability is weak. To address these limitations, recent advances such as Ensemble EMD with adaptive noise (CEEMDAN) introduce controlled perturbations to stabilize the decomposition and substantially reduce mode mixing [23], while Variational Mode Decomposition (VMD) reformulates the problem as a variational optimization that yields more stable and mathematically well-defined modes [24]. A further consideration is the challenge of performing group-level EMD analysis. Because IMFs generated by standard EMD are not guaranteed to be aligned across subjects, additional strategies are required for cross-subject comparability. Common approaches include matching IMFs based on dominant frequency bands, correlational similarity of intrinsic mode patterns, or using multivariate extensions such as Multivariate EMD (MEMD) [25], which jointly decomposes multiple time series to enforce IMF correspondence. These methods are increasingly used in resting-state and task-based fMRI studies to ensure that IMFs reflect comparable neural processes across participants. Finally, to provide a more balanced perspective, we expanded the empirical literature on EMD-based fMRI analysis, including applications in dynamic functional connectivity, identification of frequency-specific biomarkers in neurological disorders, and characterization of scale-dependent neural oscillations. Together, these additions present a more comprehensive and nuanced understanding of EMD, its limitations, recent methodological improvements, and its role in group-level fMRI analysis.

As an adaptive signal decomposition method, EMD decomposes a signal into multiple intrinsic mode functions (IMFs) with distinct frequency characteristics, thereby effectively capturing its nonlinear and nonstationary properties. This adaptability makes EMD particularly flexible for processing fMRI data acquired from different individuals or under varying experimental conditions. A key advantage of EMD is that it does not rely on strong a priori assumptions about the data or require extensive preprocessing steps, such as strict stationarity assumptions or predefined basis functions, to yield meaningful decompositions. As a result, EMD is applicable to a wide range of data types encountered in neuroimaging studies. Moreover, EMD provides frequency-resolved information for each IMF, which is essential for investigating brain activity across different frequency bands. Given that fMRI signals exhibit complex spatiotemporal dynamics, EMD facilitates the characterization of these dynamics and enables the extraction of richer, scale-dependent information. From a clinical perspective, EMD-based analyses can aid in identifying features associated with specific neurological disorders or cognitive processes, thereby offering valuable insights for clinical diagnosis and treatment planning. For example, EMD has been successfully applied in studies of Parkinson’s disease diagnosis [26] and epilepsy detection [27].

## Two-dimensional approaches

**Fig. 3:**
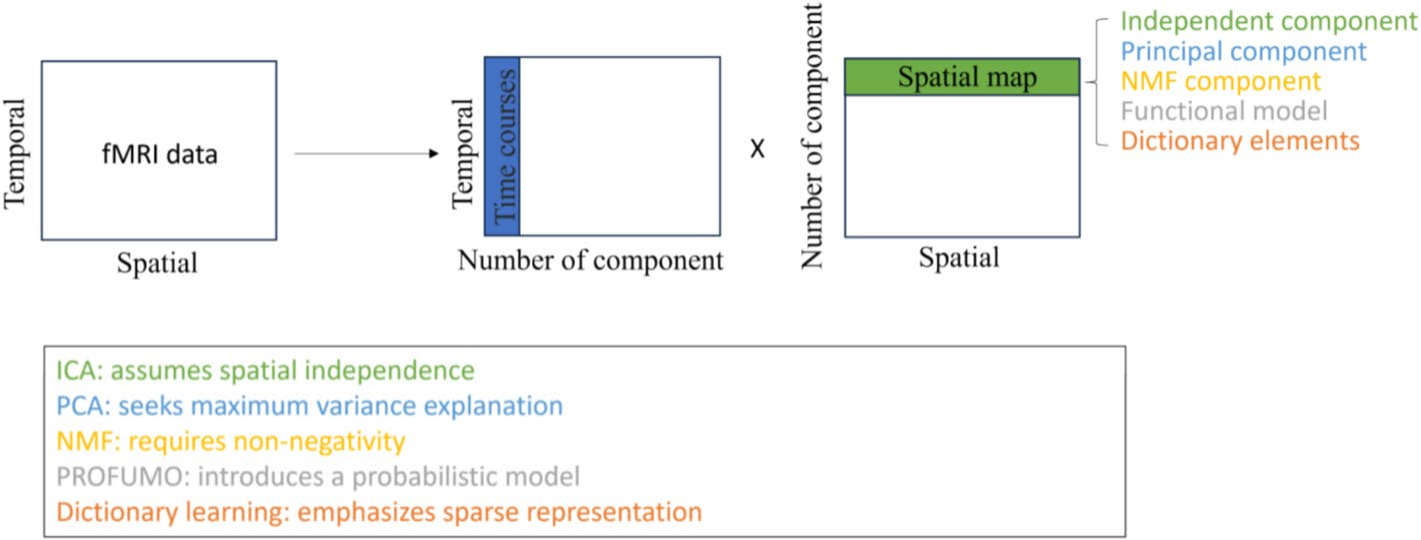
Illustration of two-way matrix decomposition. There are several techniques for two-way decomposition, each with different assumptions on the signal sources, time courses and/or spatial maps. Representative methods include ICA, PCA, NMF, PROFUMO, and Dictionary Learning

Within the category of two-dimensional approaches, as illustrated in Fig. 3, a range of matrix factorization techniques are employed to extract meaningful spatiotemporal patterns from fMRI data. Independent component analysis (ICA) and its variants, such as probabilistic ICA and guided ICA, aim to separate latent source signals by maximizing the statistical independence of the estimated components. Principal component analysis (PCA) and its extensions, including kernel PCA and sparse PCA, are widely used for linear dimensionality reduction, summarizing high-dimensional data by capturing dominant modes of variance. Probabilistic Functional Modes (PROFUMO) adopts a Bayesian hierarchical framework to infer functional patterns, enabling the simultaneous characterization of group-level commonalities and subject-specific variability in brain connectivity. Non-negative matrix factorization (NMF) imposes non-negativity constraints to produce additive and interpretable components of brain activity. Dictionary learning approaches, including semi-blind online dictionary learning, learn overcomplete representations that facilitate sparse coding of fMRI signals. Collectively, these methods constitute a versatile toolkit for investigating functional brain networks and have been widely applied in both basic neuroscience and clinical research.

### Independent component analysis

Independent component analysis (ICA) assumes that the observed data arise from linear mixtures of statistically independent latent sources, which are often interpreted as distinct brain states. These sources can be recovered through an unmixing process that maximizes the statistical independence of the estimated components [28]. ICA has been widely applied in fMRI data analysis [10, 12, 29, 30], particularly in settings where the underlying signal mixing process is unknown or difficult to model explicitly, such as unconstrained resting-state paradigms [31]. Depending on the modeling assumptions, ICA can be applied to identify either spatially or temporally independent components from fMRI data [32], and has been shown to perform effectively in both contexts when the corresponding independence assumptions are satisfied [12, 29]. In spatial ICA, independence is imposed on the spatial maps of the estimated brain states, whereas in temporal ICA, independence is enforced on their associated time courses. Detailed descriptions of the ICA algorithms, together with information on commonly used software implementations, are provided in the Appendix.

Traditional ICA algorithms face several challenges in practical applications, including sensitivity to noise and signal mixing [33], susceptibility to convergence to local optima [34], and relatively high computational demands, particularly when applied to large-scale datasets that require substantial computing resources [34]. To overcome these limitations, numerous ICA variants have been proposed. For example, bounded multivariate generalized Gaussian mixture hidden Markov model–based ICA (ICA-BMGGMM) and independent vector analysis (IVA) extend the ICA framework by incorporating bounded multivariate generalized Gaussian mixture models and hidden Markov modeling [35]. These approaches address a fundamental limitation of classical ICA, namely the strict assumption of statistical independence, which can artificially preclude overlap between functional brain networks and is inconsistent with the well-established interactive nature of neural systems. In parallel, alternative ICA algorithms such as Infomax and FastICA have been developed to improve estimation efficiency and robustness through different optimization objectives, component estimation schemes, and more flexible separation strategies [36]. Another well-known limitation of conventional ICA is the need to predefine the model order, corresponding to the number of brain networks to be estimated. Different choices of model order can yield substantially different decomposition results, posing challenges for reproducibility and interpretation [37]. To mitigate this issue, Snowball ICA [38], an iterative ICA-based approach, was introduced to enhance the stability and reliability of network estimation. Furthermore, guided ICA [39] incorporates prior information to steer the extraction of independent components, thereby improving the interpretability and reliability of the resulting decompositions. Constrained ICA (cICA) [40] similarly imposes explicit constraints or prior knowledge to enhance component separation and interpretability. Semiblind ICA [41] leverages spatial priors to improve ICA performance in fMRI analysis, particularly for estimating task-related components and identifying canonical networks such as the default mode network. Beyond linear ICA formulations, nonlinear ICA methods have also been applied to resting-state MEG data [42]. These unsupervised approaches are capable of capturing complex patterns of spontaneous cortical activity, achieving performance comparable to deep neural networks under limited-label conditions, and providing effective feature extraction and task decoding when annotated data are scarce.

From a practical perspective, the choice among different ICA variants should be guided by data characteristics and analytical objectives. Classical ICA implementations such as Infomax and FastICA are generally suitable for exploratory analyses with moderate noise levels and limited computational resources, but they may be less robust when component overlap or complex temporal dependencies are present. Variants such as ICA-BMGGMM and Independent Vector Analysis (IVA) are more appropriate when modeling interactions or overlaps between functional brain networks is of interest [43], although their increased model complexity can lead to higher computational demands. When model order uncertainty poses a concern, iterative approaches such as Snowball ICA provide improved stability and reliability [38], whereas methods requiring a fixed number of components may yield inconsistent results across different settings. Guided ICA [39], constrained ICA (cICA) [40], and semiblind ICA [41] are particularly advantageous in scenarios where reliable prior information is available, such as task-based fMRI or hypothesis-driven studies, but they may introduce bias if the imposed priors are inappropriate or overly restrictive. For resting-state MEG or other highly nonlinear neural signals, nonlinear ICA methods are better suited to capture complex spontaneous activity patterns, while linear ICA variants may fail to adequately model such dynamics. Overall, no single ICA variant is universally optimal, and mismatches between model assumptions and data properties should be avoided to ensure robust and interpretable results.

ICA offers several notable advantages for the analysis of fMRI data. It is capable of identifying brain activation patterns, separating signals originating from different sources, characterizing underlying data structure, and revealing correspondences between spatial maps and temporal dynamics, while also facilitating denoising to isolate meaningful neural components [44]. By adjusting the model order, ICA can extract brain network components at different spatial scales [45]. When a relatively large model order is selected (e.g., greater than 100), the resulting components can approximate fine-grained brain parcellations [46]. ICA has also demonstrated substantial utility in clinical applications. In disease diagnosis and classification, ICA-based analyses of patient fMRI data can identify abnormal functional patterns associated with specific neurological and psychiatric conditions, contributing to early detection and differential diagnosis of disorders such as Alzheimer’s disease (AD) [47], schizophrenia (SZ), and bipolar disorder (BP) [44]. During treatment and intervention, ICA can be used to assess therapeutic effects [48], monitor changes in brain function and recovery processes, and inform the adjustment and optimization of treatment strategies [49]. Overall, ICA provides a powerful and flexible framework for investigating the functional organization and dynamic activity of the brain, supporting both fundamental neuroscience research and clinical applications.

### Principal component analysis

Principal component analysis (PCA) is a classical data analysis technique that dates back to the early twentieth century. It was originally introduced by Karl Pearson in 1901 [50] and subsequently formalized by Harold Hotelling in 1933 [51]. The central objective of PCA is to project high-dimensional data into a lower-dimensional subspace, capturing the dominant modes of variance while reducing noise and redundancy. Through this linear transformation, PCA simplifies complex datasets while preserving as much of the variance in the original data as possible. In neuroscience research, PCA was first applied to fMRI data in [12], where it was used to transform high-dimensional spatiotemporal signals into a compact set of features. This dimensionality reduction facilitates computational efficiency and provides a clearer representation of large-scale patterns in brain activity. The theoretical foundation of PCA is based on the eigendecomposition of the data covariance matrix or, equivalently, singular value decomposition (SVD) [52]. In the context of fMRI analysis, PCA typically involves constructing a data matrix from preprocessed fMRI signals, followed by applying SVD to extract principal components that characterize the dominant patterns of variation and functional activity across brain regions. Detailed descriptions of the PCA algorithm, together with information on commonly used software implementations, are provided in the Appendix.

Traditional PCA is based on the assumption that the underlying data structure can be adequately captured through linear combinations of variables. However, many real-world datasets, including fMRI data, exhibit pronounced nonlinear characteristics. To address this limitation, kernel PCA was introduced as a nonlinear extension of PCA [53]. By applying kernel functions, kernel PCA implicitly maps the data into a high-dimensional feature space, enabling the extraction of principal components that better capture nonlinear structure. Importantly, this mapping allows the principal components in the feature space to be computed efficiently without explicitly performing the high-dimensional transformation. To improve the interpretability of PCA results, Zou, Hastie, and Tibshirani proposed sparse PCA in 2006 [54], which introduces sparsity constraints such that many loadings in the principal components are forced to zero. This sparsity facilitates more interpretable representations by highlighting a limited set of contributing variables. In addition, incremental PCA [55], an extension of classical PCA, enables efficient processing of large-scale datasets by updating principal components sequentially, thereby reducing memory requirements and computational burden. When applied to large multi-subject fMRI datasets, standard PCA-based analyses can encounter substantial scalability challenges due to the size of aggregated data. To overcome these issues, Stephen M. Smith et al. proposed two group-level PCA approaches in 2014: MIGP (Incremental Group PCA of MELODIC) and SMIG (Small Memory Iterative Group PCA). These methods were designed to be more robust and memory-efficient than conventional approaches for analyzing multi-subject resting-state fMRI data [56]. The resulting group-level PCA outputs can subsequently be used in downstream analyses, including estimation of group-average voxel connectivity, group-level parcellation, and group ICA. Building on this work, Vince D. Calhoun et al. reviewed group-level PCA methods in 2015 and proposed further corrections and improvements to memory-efficient PCA strategies, thereby enhancing the scalability and effectiveness of group ICA for large datasets [57]. Collectively, these PCA variants address different practical challenges and offer flexible solutions tailored to diverse data characteristics and analysis goals.

From a practical perspective, the choice among different PCA variants should be guided by data structure, scale, and analysis objectives. Classical PCA is well suited for linear dimensionality reduction and noise suppression in relatively low-dimensional or moderately sized datasets, but it is limited in capturing nonlinear relationships and may therefore be inappropriate for data with complex manifold structures. In such cases, kernel PCA provides a more suitable alternative by modeling nonlinear dependencies, although its computational and memory requirements can become prohibitive for large-scale fMRI datasets. Sparse PCA is particularly advantageous when interpretability is a primary concern, as it yields components with localized spatial support. However, overly strong sparsity constraints may discard meaningful distributed patterns and reduce robustness in low signal-to-noise settings. Incremental PCA and its group-level extensions, such as MIGP and SMIG, are specifically designed for large multi-subject fMRI datasets, where memory efficiency and scalability are critical. These methods are therefore preferable to standard PCA in large-scale or population-level analyses, while providing limited benefits for small datasets where computational resources are not constrained. Overall, selecting an appropriate PCA variant requires balancing linearity assumptions, interpretability, computational efficiency, and dataset size to avoid mismatches between model capabilities and practical requirements.

In contemporary brain state research, PCA is widely adopted due to its conceptual simplicity and effective dimensionality reduction capability. First, as a general linear dimensionality reduction technique, PCA is well suited for the integration of multimodal data. When applied to multimodal datasets, PCA can identify dominant patterns of shared variance across modalities, thereby facilitating a more comprehensive understanding of brain structure and function. For example, in studies combining data from different imaging modalities—such as fMRI, structural MRI (sMRI), and EEG—PCA can help uncover relationships between modalities, extract common information, and characterize the multifaceted nature of brain activity [58]. Second, the linear transformation and computational efficiency of PCA make it particularly attractive for real-time and dynamic analyses. In real-time fMRI and dynamic network studies, PCA can rapidly capture major variations in the data, allowing principal components to be updated efficiently as new data become available. This property supports analyses with relatively high temporal resolution and enables the tracking of evolving brain activity patterns over time [59]. Finally, PCA has shown promise in the context of personalized and precision medicine. By capturing the dominant sources of variance in neuroimaging data, PCA can help reveal individual differences in brain connectivity and activity patterns, thereby supporting personalized diagnostic and therapeutic strategies. For instance, PCA has been used to identify multivariate brain signatures associated with complex conditions such as bipolar disorder and obesity [60], as well as to characterize principal components of whole-brain phase synchronization in autism spectrum disorder [61]. More broadly, PCA facilitates the extraction of both shared and individual-specific features from high-dimensional neuroimaging data, providing a quantitative basis for personalized prognosis prediction in stroke patients [62] and for the clinical diagnosis of mild to moderate Alzheimer’s disease [63].

### Probabilistic functional modes

Probabilistic Functional Modes (PROFUMO) was introduced by Harrison et al. in 2015 as a probabilistic modeling framework for fMRI data analysis [64]. PROFUMO was developed to address limitations of traditional decomposition methods, which often struggle to adequately capture complex functional connectivity patterns and heterogeneous activity across distributed brain regions. By adopting a probabilistic formulation, PROFUMO enables a more flexible and principled characterization of fMRI data through explicit parameter estimation and uncertainty modeling, thereby facilitating the investigation of coordinated activity among brain regions. At its core, PROFUMO employs a hierarchical Bayesian model to analyze resting-state fMRI data. Within this framework, the observed fMRI signals are modeled as the product of spatial maps and corresponding temporal processes, with additive noise explicitly accounted for. Model parameters are estimated using variational Bayesian inference, and spatial effects are characterized using a Delta–Gaussian mixture model. In addition, PROFUMO incorporates correlations induced by the hemodynamic response function (HRF) to model the temporal autocorrelation structure of the BOLD signal. Through this probabilistic and hierarchical approach, PROFUMO can efficiently process large-scale fMRI datasets, identify both group-level functional commonalities and subject-specific variations, and characterize dynamic changes in functional connectivity. Detailed algorithmic descriptions of PROFUMO, together with information on readily available software implementations, are provided in the Appendix.

Building on the PROFUMO framework, Farahibozorg et al. introduced sPROFUMO in 2021 as a scalable extension designed for large-scale fMRI analyses [65]. sPROFUMO formulates a hierarchical model of spatial topology and functional connectivity, enabling bottom-up, data-driven estimation of group-level functional patterns. These group-level estimates are subsequently used in a top-down manner to regularize subject-specific representations, allowing the model to accommodate substantial inter-subject heterogeneity. A primary motivation for sPROFUMO is to alleviate the computational burden associated with bidirectional hierarchical modeling in large datasets. By introducing algorithmic simplifications and more efficient inference strategies, sPROFUMO substantially improves computational efficiency and scalability compared with the original PROFUMO framework. In addition, sPROFUMO has demonstrated strong predictive capability with respect to individual cognitive variation, showing robust associations with a range of cognitive measures, including sensorimotor performance, memory, executive function, and general fluid intelligence. Owing to its scalability and predictive relevance, sPROFUMO provides a practical and powerful tool for population-level studies of functional brain organization.

From a practical standpoint, the choice between PROFUMO and its scalable extension sPROFUMO should be guided primarily by dataset size, computational resources, and analysis goals. Traditional PROFUMO is well suited for studies with moderate sample sizes where detailed probabilistic modeling of spatial and temporal variability is desired, but its bidirectional hierarchical inference can become computationally prohibitive for large-scale datasets. In contrast, sPROFUMO is specifically designed for large population-level fMRI studies, where scalability and computational efficiency are critical, making it preferable for applications involving thousands of subjects or predictive modeling of cognitive traits. However, the simplified inference strategy employed by sPROFUMO may be less advantageous in small-sample or hypothesis-driven studies that require fine-grained modeling of individual-level spatial heterogeneity. In such scenarios, the full PROFUMO framework may provide more detailed subject-specific representations at the cost of increased computational burden. Therefore, the selection of PROFUMO-based variants should balance modeling fidelity against scalability requirements to avoid unnecessary computational overhead or loss of representational detail.

By explicitly decomposing fMRI data into spatial and temporal components within a probabilistic framework, PROFUMO is well suited for handling complex spatiotemporal patterns. Through probabilistic modeling, the method can uncover latent and subtle functional connections that may be difficult to detect using deterministic approaches, thereby increasing the potential for identifying previously unrecognized functional relationships in the brain. Empirical studies have demonstrated that, compared with traditional methods such as ICA, PROFUMO is more effective at disentangling complex patterns of brain activity, offering deeper insights into functional networks and their interactions. A key strength of PROFUMO lies in its explicit modeling of inter-subject variability. By allowing spatial maps to vary across individuals while constraining them through a hierarchical probabilistic structure, PROFUMO captures subject-specific differences in functional topography rather than enforcing identical spatial patterns across all participants. This flexibility is particularly advantageous in studies involving heterogeneous populations or group comparisons, as it provides a more interpretable account of how and why functional connectivity patterns differ between individuals. Finally, the introduction of stochastic variational Bayes inference substantially reduces the computational burden of PROFUMO, lowering the original large-scale computational requirements by orders of magnitude. This improvement enables the efficient analysis of large datasets, such as those from the Human Connectome Project and the UK Biobank, thereby facilitating more comprehensive investigations of large-scale functional organization and variability in the human brain.

Overall, PROFUMO’s versatility and its ability to analyze complex fMRI data make it a valuable tool for neuroscience research. In clinical studies, PROFUMO can be used to investigate brain abnormalities and to identify biomarkers associated with a range of neurological and psychiatric disorders, thereby facilitating understanding of the underlying neural mechanisms and potential targets for therapeutic intervention. For example, PROFUMO has been applied to examine changes in brain function and structure induced by long-term alcohol consumption, as well as the contribution of visual networks to the psychopathology of depression [66]. In addition, PROFUMO has been employed to study age-related changes in brain development and functional connectivity [67], providing insights into the evolution of brain functional organization across the lifespan. sPROFUMO is designed for large-scale data analysis [65] and is capable of processing large datasets while reducing computational costs.

### Nonnegative matrix factorization

Nonnegative matrix factorization (NMF), originally proposed in 1999 [68], is a matrix factorization and feature extraction technique that is particularly well suited to data subject to nonnegativity constraints. NMF was initially developed for the analysis of image data through component-based decomposition, with the goal of representing complex data by learning parts-based representations of objects. Over time, and with continued methodological advances, NMF has been widely adopted across a variety of application domains. In neuroscience, Lohmann, Volz, and Ullsperger first applied NMF to fMRI data analysis in 2007 [13]. In their study, NMF was introduced as a novel approach for analyzing single-trial fMRI data. Specifically, the fMRI data from each trial are represented as a matrix in which rows correspond to time points and columns correspond to the spatial distribution of brain states. Using NMF, these matrices are decomposed into weighted linear combinations of nonnegative components (basis functions). These basis functions emphasize salient features of the dataset and facilitate characterization of trial-specific response patterns. Moreover, analysis of the basis functions obtained via NMF enables investigation of the response characteristics of different brain regions within a single trial, as well as potential interactions among these characteristics across regions. Finally, clustering techniques were applied to the NMF-derived components to identify and interpret functional connectivity patterns and underlying neural mechanisms across trials. The detailed algorithmic formulation of this approach, together with information on readily available software implementations, is provided in the Appendix.

In addition to the classical NMF algorithm, numerous variants and extensions have been developed to accommodate different data characteristics and analytical requirements. For example, sparse NMF [69] incorporates sparsity constraints, encouraging most elements of the basis vectors and coefficient matrices to be zero. Traditional NMF typically assumes that the input data are unlabeled, however, in many practical scenarios, partial label information may be available and can be exploited. Semi-supervised NMF [70] integrates both labeled and unlabeled data to improve the performance and interpretability of the factorization. Beyond sparse and semi-supervised formulations, other extensions include structured NMF [71] and deep NMF inspired by deep learning architectures [72]. These approaches further broaden the applicability of NMF and enable adaptation to more diverse analytical needs. Concurrently, an increasing number of studies have investigated the application of NMF to fMRI data analysis. For instance, NMF-based methods have been employed to study adult attention-deficit/hyperactivity disorder, including a constrained NMF algorithm based on α-divergence [73]. Additionally, a spatially constrained NMF approach has been proposed for detecting BOLD signals in fMRI data [74]. To estimate task-related neuronal activity from fMRI measurements, researchers have also evaluated the performance of various NMF algorithms, such as alternating least squares NMF [75] and NMF formulations based on divergence-based cost functions [76].

From an application-oriented perspective, the selection of NMF variants should be driven by data availability, interpretability requirements, and prior knowledge. Sparse NMF is particularly suitable when interpretability and localized activation patterns are of primary interest, as the imposed sparsity constraints yield components with clearer spatial structure. However, excessive sparsity may suppress distributed brain networks and reduce robustness in low signal-to-noise fMRI data. Semi-supervised NMF is advantageous when partial label information is available, such as in task-based or clinical classification studies, but its performance may degrade when labels are unreliable or inconsistent. Structured and spatially constrained NMF variants are preferable when anatomical or functional priors are available, as they improve the physiological plausibility of the extracted components. In contrast, these constraints may introduce bias if prior assumptions are misspecified. Deep learning–based NMF methods provide greater representational flexibility and can capture complex nonlinear patterns, but they typically require larger sample sizes and increased computational resources, making them less suitable for small-scale studies. Overall, mismatches between constraint strength, data quality, and study objectives should be avoided to ensure stable and interpretable NMF-based decompositions in fMRI analysis.

NMF has been widely applied in neuroimaging because its non-negativity constraint naturally yields parts-based and additive representations [69, 77, 78]. This constraint is particularly suitable for data modalities in which the underlying generative process is inherently non-negative and additive, such as gray-matter density, white-matter tissue profiles, activation amplitude maps, or other energy-like voxelwise measures. In these cases, NMF can effectively reduce a common form of signal mixing in which positive and negative contributions from different latent sources cancel each other out, producing components that are difficult to interpret. By enforcing non-negativity, NMF avoids such cancellation effects and yields spatial components that more directly reflect additive contributions from localized brain regions, thereby enhancing interpretability. However, non-negativity does not prevent all types of signal mixing and is not universally appropriate for all neuroimaging data. When the true sources include meaningful negative fluctuations—such as task-evoked deactivations, anticorrelated functional networks, or signed spatial patterns commonly observed in ICA—the imposed non-negative constraint may not align with the underlying data-generating process. In particular, raw BOLD time series and many spatial maps naturally contain both positive and negative variations arising from neural dynamics, physiological noise, baseline drifts, and preprocessing transformations. In such scenarios, enforcing non-negativity may distort the intrinsic signal structure and bias the recovered components, potentially exacerbating rather than alleviating signal mixing. Therefore, the suitability of NMF depends critically on whether the underlying neural or structural processes can be reasonably modeled as additive and non-negative. Although brain activity may involve complex and potentially nonlinear interactions whose exact form remains unknown, the non-negativity constraint can still promote more interpretable component representations when it is consistent with the physical meaning and statistical properties of the data modality.

In medical diagnosis, NMF has been applied to the analysis of fMRI data to identify abnormal brain functions associated with specific diseases. For example, one study employed NMF to uncover aberrant brain patterns related to anxiety recognition, thereby contributing to the development of more informed diagnostic and treatment strategies [79]. Moreover, NMF facilitates investigation of disease mechanisms by identifying characteristic neural activity patterns, and has been used in the study and diagnosis of disorders such as schizophrenia and depression [80]. With respect to brain function reconstruction, NMF can assist in restoring functional connectivity in patients with brain damage and may support rehabilitation efforts [81]. For instance, analyses of interdependence across trials and brain regions [13] illustrate the potential of NMF-based approaches in this domain.

### Dictionary learning

Dictionary learning originated in the 1990s. In 1993, Mallat and Zhang published a groundbreaking paper [82], which introduced the concept of an overcomplete dictionary and proposed a matching pursuit algorithm for solving sparse representation problems under such dictionaries. Subsequently, Chen et al. proposed a basis pursuit method for solving sparse optimization problems in 1998 [83]. The field gained widespread attention following the publication of a pioneering paper on sparse coding of natural images by Olshausen et al. in 1996 [84, 85]. Over time, dictionary learning techniques have been adapted for fMRI data analysis. For instance, in 2006, M. Aharon et al. proposed the K-SVD algorithm based on sparse dictionary learning [86]. K-SVD is a sparse coding process that constructs a dictionary matrix by learning a set of overcomplete basis vectors from a sample set, where each basis vector corresponds to a column of the dictionary. Any sample can then be represented sparsely using this dictionary. In 2019, semi-blind online dictionary learning (Semi-ODL) [87] further advanced the performance of dictionary learning methods. The detailed algorithmic formulation of this approach, along with information on readily available software implementations, is provided in the Appendix.

Semi-ODL outperforms methods such as OLD and Sparse Dictionary Learning (SDL). Notably, it demonstrates higher computational efficiency than ODL and SDL. Semi-ODL also exhibits strong spatial detection capability and provides effective estimation of temporal processes, particularly in separating task-related components from multi-task fMRI data. The method enhances the dictionary learning process by incorporating temporal prior information from the task paradigm into the ODL framework, thereby improving the extraction performance of task-related components. Furthermore, Semi-ODL allows flexible adjustment of two key parameters: the tolerance value (t) and the correction factor (e). Tuning these parameters controls the constraint weights imposed on dictionary atoms and determines the required degree of correction, enabling customization for specific fMRI datasets to achieve improved results.

In implementing dictionary learning, several hyperparameters must be determined, including the sparse regularization coefficient (λ), the dictionary size (i.e., the number of atoms), and the stopping criterion. The hyperparameter λ is typically selected through empirical tuning within a small grid to achieve an appropriate balance between sparsity of the coefficient matrix and reconstruction accuracy. The number of atoms is commonly set to slightly exceed the expected number of meaningful spatial components, providing sufficient representational flexibility while reducing the risk of overfitting. Convergence is generally assessed using a stopping criterion based on the relative decrease of reconstruction error (e.g., < 1e–4) or by specifying a maximum number of iterations (such as 200). These strategies reflect widely adopted practices in fMRI-related dictionary learning studies and offer a stable, reproducible configuration for practical applications.

Dictionary learning holds considerable potential for clinical applications, particularly in the analysis of complex fMRI data. For instance, it has been successfully employed to identify distinct functional subnetworks in resting-state brain connectivity [88] and to classify and segment brain tumors [89]. Furthermore, dictionary learning has been utilized for classifying individuals with Alzheimer’s disease, mild cognitive impairment, and those who are cognitively intact [90].

## Multi-dimensional apporaches

**Fig. 4:**
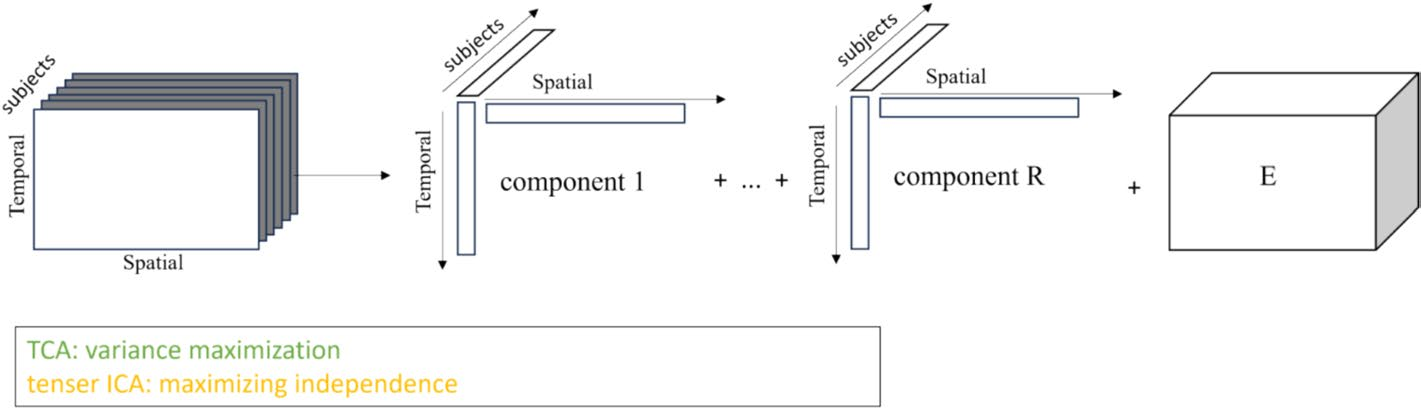
Illustration of tensor decomposition, shown here using a third-order tensor as an example. This paper focuses on CPD-based models, including TCA and SNTD. CPD represents a tensor as a sum of multiple rank-one components plus a residual term, enabling joint modeling of multi-subject or multi-modal fMRI data

Within the scope of Multi-Dimensional Approaches, this paper highlights three key tensor-based methods for their ability to decompose complex fMRI data across spatial, temporal, and subject/session domains. Figure 4 illustrates an example of third-order tensor decomposition. Tensor ICA leverages statistical independence to separate mixed signals into distinct sources, while its probabilistic extension, Tensor PICA, enhances robustness by explicitly modeling noise. In contrast, TCA employs a constraint-free Canonical Polyadic Decomposition to uniquely extract shared spatiotemporal patterns across subjects, making it particularly suitable for naturalistic stimuli studies. Finally, SNTD incorporates non-negativity and sparsity constraints to isolate frequency-specific co-activation patterns, demonstrating its value in identifying biomarkers for neurological disorders such as Parkinson's disease. Collectively, these techniques exploit the multi-way structure of the data to enhance the interpretability and robustness of functional brain network analysis.

### Tensor independent component analysis

Beckmann and Smith explored a method for analyzing fMRI data from multiple subjects or sessions in 2005 [15]. Specifically, their study was based on probabilistic independent component analysis (PICA), which provides a probabilistic formulation of ICA within a maximum-likelihood framework. While classical ICA can, in principle, be formulated with a general multi-parameter noise covariance matrix that allows non-spherical or correlated noise structures, PICA typically adopts a more restrictive noise model by assuming isotropic Gaussian noise with covariance of the form σ^2^I. This simplification facilitates analytical tractability and parameter identifiability but limits the ability to model more complex noise covariance patterns that may be present in fMRI data. As a result, although ICA and PICA share similar generative formulations, they differ substantially in their assumptions about noise structure, with ICA offering greater generality and PICA emphasizing computational feasibility under constrained noise assumptions. Building on the PICA framework, Beckmann and Smith further proposed tensor probabilistic independent component analysis (Tensor PICA) by extending the model to higher dimensions. Tensor PICA performs a tensor decomposition of preprocessed fMRI data to estimate spatially and temporally independent components. During the decomposition, additional constraints based on maximum non-Gaussianity are imposed on the spatial maps to enhance source independence, leading to a more robust and interpretable characterization of activity patterns in fMRI data. The resulting optimization problem is typically solved by maximizing an objective function that enforces non-Gaussianity, often within a maximum-likelihood estimation framework. The detailed algorithmic formulation of this method, along with information on readily available software implementations, is provided in the Appendix.

As methodologies have advanced, Tensor PICA extends Tensor ICA by incorporating a probabilistic modeling framework, which enhances the robustness and reliability of signal separation through explicit noise modeling. As a generalization of the single-session probabilistic independent component analysis model, Tensor PICA offers the added advantage of multidimensional decomposition. Research indicates that the Tensor PICA method effectively extracts signal components across spatial, temporal, and subject dimensions, demonstrating strong performance in both simulated and real fMRI data applications. Furthermore, Tensor PICA can more accurately model complex data structures, particularly in studies involving multi-session or multi-subject designs, thereby contributing to a more comprehensive understanding of functional brain imaging data. Clinically, this method has been employed in numerous studies, including investigations into the maintenance of cognitive performance in relapsing–remitting multiple sclerosis (RRMS) [91], analyses of neurofeedback regulation and recovery processes [92], and examinations of the social and reward networks associated with major depressive disorder [93].

### Tensor component analysis

Tensor Component Analysis (TCA), also known as CANDECOMP/PARAFAC (CP) decomposition [94], is a fundamental model for decomposing multi-dimensional data with more than two dimensions. Since fMRI signals are inherently multidimensional, they can be naturally represented in tensor form. TCA was developed to extract meaningful brain activity patterns from complex, high-dimensional functional magnetic resonance imaging datasets. It addresses heterogeneity and variations across multiple data domains, thereby enhancing the performance of cross-domain data analysis and related applications. Unlike tensor ICA, TCA imposes no constraints on the decomposition. The detailed algorithmic formulation of this method, along with information on readily available software implementations, is provided in the Appendix.

TCA can effectively extract common features from data. It is particularly suitable for analyzing naturalistic fMRI data, as the method naturally aligns with the brain state characteristics evoked by naturalistic stimuli across different subjects. Consequently, it yields consistent time series and spatial distribution maps, thereby enhancing analytical precision. This capability allows TCA to effectively capture shared features and reveal functional brain connectivity and network structures under naturalistic conditions. These advantages establish TCA as a powerful tool for processing and analyzing fMRI data, providing deeper insights into brain function and assisting researchers in better understanding the brain's spatiotemporal dynamics and functional connectivity.

Overall, the TCA algorithm holds significant potential for both fMRI research and clinical applications. fMRI data provide valuable information about brain function and structure, offering potential utility for disease diagnosis and monitoring. For example, TCA has been applied to the study of Alzheimer's disease [95]. Furthermore, TCA can assist in identifying functional or structural brain alterations to evaluate treatment efficacy and guide subsequent therapeutic strategies. This includes, for instance, assessing brain states through naturalistic stimulation paradigms [14].

### Sparse nonnegative tensor decomposition

Sparse Nonnegative Tensor Decomposition (SNTD) [16] was initially developed to investigate frequency-specific dynamic changes in brain activity across different frequency bands in patients with Parkinson's disease (PD) and to estimate frequency-specific co-activation patterns (CAPs). SNTD estimates frequency-specific CAPs more effectively than traditional CAP methods and reveals significant differences in PD patients. Within fMRI research, the SNTD method can be employed to study frequency-specific alterations in brain activity and to explore functional connectivity patterns across distinct frequency bands. By applying SNTD to fMRI data, researchers can gain deeper insights into differences in brain activity between individuals with Parkinson's disease and healthy controls, thereby elucidating the pathophysiology of PD. The detailed algorithmic formulation of this method, along with information on readily available software implementations, is provided in the Appendix.

In clinical applications, the SNTD method may contribute to the identification of early biomarkers for neurological disorders such as Parkinson's disease and Alzheimer's disease [16]. By analyzing frequency-specific dynamic changes in patient fMRI data, SNTD can detect physiological and functional alterations associated with disease at an earlier stage with greater sensitivity. Furthermore, the SNTD method can be employed to evaluate the efficacy of treatments for neurological disorders. This assessment is achieved by comparing patient fMRI data before and after treatment and analyzing frequency-specific changes in brain activity. Overall, SNTD provides a novel approach for studying dynamic connectivity changes in brain networks through the extraction of frequency-specific CAPs.

## Discussion

In this review, we provide a comprehensive overview of decomposition methods commonly used for estimating brain states from fMRI data. Our survey encompasses classical matrix-based techniques—including PCA, ICA, NMF, dictionary learning, and their probabilistic or sparsity-constrained variants—as well as modern tensor-based approaches such as Tensor ICA, TCA, and SNTD. For each method, we describe its mathematical foundations, algorithmic implementation, and practical considerations, including model-order selection, reproducibility, and interpretability. By systematically examining these methods and their variants, this review aims to offer readers a thorough understanding of the current landscape of brain-state decomposition techniques and to highlight their potential applications in cognitive neuroscience and clinical research.

While each method offers unique advantages, no single approach is universally optimal. For instance, PCA is well-suited for dimensionality reduction but may fail to disentangle biologically meaningful networks, whereas ICA provides higher interpretability for individual network structures but can be sensitive to noise and requires careful model-order selection. Tensor-based methods such as TCA and Tensor ICA offer the ability to simultaneously model multi-subject data, capturing inter-subject variability, but their computational cost and identifiability constraints must be carefully considered. Sparsity and probabilistic modeling, as in NMF, SNTD, and PROFUMO, can improve robustness and interpretability, yet also introduce additional hyperparameters that need tuning based on dataset characteristics. Furthermore, nonlinear extensions of traditional methods, including kernel PCA [53], kernel ICA [96], and kernel NMF [97], provide an avenue for capturing complex, non-linear brain activity patterns that may be missed by linear decompositions. These approaches, however, come with higher computational demands and require careful kernel selection. By synthesizing these considerations, researchers can make informed decisions about which decomposition approach is most suitable for their specific experimental design, sample size, and desired level of interpretability. We envision that future work will increasingly integrate kernel-based, probabilistic, and tensor methods to leverage their complementary strengths, improving the precision and robustness of brain state estimation in fMRI studies. To provide a concise comparison of these methods across key dimensions, we summarize their assumptions, dimensionalities, constraints, noise models, computational costs, reproducibility, ability to reduce state mixture, robustness on small datasets, handling subject variance, clinical applicability, biological explainability and interpretability implications in Supplementary Table 1.

In tensor-based fMRI decomposition, CPD and PARAFAC2 [98] provide foundational frameworks that underpin methods such as TCA, Tensor ICA and SNTD. CPD decomposes a multi-way tensor into a sum of rank-1 components and ensures uniqueness under mild Kruskal-rank conditions [99], offering formal identifiability guarantees essential for multi-subject analyses. PARAFAC2 relaxes the strict assumptions of CPD by allowing one mode—typically the temporal mode—to vary across subjects or sessions, enabling alignment of longitudinal or multi-subject data while preserving interpretability of the extracted components. In contrast, less constrained tensor models such as Tucker or SNTD provide flexible representations with fewer uniqueness guarantees, trading off interpretability for modeling flexibility. Considering these properties, researchers can select tensor decomposition approaches that balance identifiability, interpretability, and flexibility depending on the experimental design, number of subjects, and the nature of temporal or longitudinal variability.

A meaningful comparison between decomposition methods requires not only understanding their mathematical formulations but also the metrics used to evaluate their performance. Quantitative and qualitative evaluation metrics are essential for assessing whether a decomposition yields reliable, biologically meaningful components. Stability-based metrics provide an important dimension of evaluation: methods such as ICASSO [100], bootstrapping, or split-half reproducibility assess how consistently components are recovered across repeated runs or dataset partitions, offering insights into algorithmic robustness. Reconstruction-based metrics, including reconstruction error, explained variance, and cross-validated prediction accuracy, quantify how well the decomposition captures the underlying signal structure. Beyond algorithmic performance, biological validity can be examined through cross-modal reproducibility (e.g., correspondence between fMRI networks and structural connectivity, EEG/MEG networks, or behavioral profiles) or comparison across independent datasets. Together, these evaluation metrics allow researchers to systematically assess the strengths and limitations of different decomposition approaches and to select methods that best align with their analytical goals.

Computational considerations are critical when applying decomposition methods to large-scale fMRI datasets. Matrix-based techniques such as PCA and ICA are generally efficient for datasets of moderate size but can become memory-intensive when applied at the voxel level across hundreds of subjects. Tensor-based approaches, including TCA, Tensor ICA, and SNTD, enable simultaneous modeling of multi-subject or multi-session data, capturing inter-subject variability more effectively, but at the cost of increased runtime and memory usage. Sparse and probabilistic methods, such as NMF, SNTD, and PROFUMO, involve iterative optimization and additional hyperparameters, which further amplify computational demands. To address these challenges, practical strategies include reducing dimensionality prior to decomposition, using batch-wise computations, leveraging GPU acceleration, and employing parallel processing frameworks. By considering these factors, researchers can balance methodological rigor with computational feasibility, ensuring that large-scale and high-dimensional fMRI analyses remain tractable while preserving interpretability and robustness of the extracted brain states.

To address common methodological considerations across decomposition frameworks, we further compare the major algorithms along several key neuroimaging criteria, including biological interpretability, robustness on small datasets, state-mixture reduction, handling of subject-level variability, clinical applicability, and reproducibility. ICA generally provides the strongest biological interpretability because its independence assumption yields spatially meaningful and physiologically distinct networks, whereas PCA—although valuable for dimensionality reduction—does not perform true source separation and therefore produces components that are less physiologically specific. NMF yields parts-based representations that enhance interpretability but may suffer from instability on small samples. Tensor-based models such as CPD/TCA reduce state mixture more effectively by maintaining multi-way structure and can better capture subject variance through shared–individual factorization. Bayesian approaches such as PROFUMO additionally incorporate hierarchical priors to model inter-subject variability, providing advantages in clinical applications requiring stable cross-site reproducibility. To make these comparisons explicit, we added a summary table in the Supplementary Table 1 that evaluates each method across these commonly used neuroimaging criteria and highlights the scenarios in which each method is most appropriate.

Although the reviewed data decomposition methods differ substantially in their mathematical formulations, they share several common principles regarding hyperparameter tuning. For linear decomposition approaches such as PCA and ICA, the primary hyperparameters typically involve the number of components, which is often determined using variance-explained criteria, information-theoretic measures, or stability-based analyses. In contrast, methods incorporating sparsity or non-negativity constraints, including NMF, dictionary learning, and sparse nonnegative tensor decomposition, require careful selection of regularization strengths to balance reconstruction accuracy and interpretability, commonly guided by cross-validation, sparsity metrics, or prior neurophysiological knowledge. Probabilistic frameworks such as PROFUMO introduce additional hyperparameters related to prior distributions and model complexity, which are usually tuned via Bayesian evidence maximization or empirical Bayes strategies. For multivariate and tensor-based methods, including tensor PICA and tensor component analysis (TCA), hyperparameter selection often involves jointly determining model rank along multiple modes, with sensitivity analyses and reproducibility across subjects serving as practical evaluation criteria. Finally, adaptive and data-driven approaches such as EMD typically rely on signal-driven stopping criteria rather than explicit hyperparameter tuning. Collectively, these strategies highlight that effective hyperparameter tuning in fMRI data decomposition is application-dependent and benefits from a combination of data-driven validation, model stability assessment, and domain-specific prior knowledge.

### Model order selection

Model order selection (MOS) is a common challenge in data decomposition methods, particularly when dealing with high-dimensional data. It involves determining the optimal number of source signals—a decision that critically influences the outcomes of data processing. Selecting an appropriate model order ensures that the model is sufficiently flexible to capture the complexity of the data structure while avoiding overfitting or underfitting. An inappropriate model order can lead to substantially divergent results even when using the same dataset. Taking ICA as an example, Allen and colleagues demonstrated that an insufficient model order results in distorted spatial distributions of the components estimated by ICA. However, increasing the model order beyond the true number of sources may cause some brain networks to split into multiple independent components [101].

In practical applications, an appropriate model order enables an algorithm to reliably separate and reconstruct source signals from observed mixtures, thereby enhancing the accuracy and robustness of neuroimaging decomposition methods. Selecting the optimal model order involves balancing model complexity with data representation fidelity to ensure that the results are both reliable and interpretable.

In fMRI data processing, the performance of decomposition methods directly influences the effectiveness of downstream analyses. Selecting an appropriate model order is therefore critical for enhancing the performance and reliability of these analytical tasks. However, determining the model order remains a complex and challenging problem in fMRI data analysis. First, raw fMRI data do not directly provide an approximate range for the number of source signals. This is because functional networks are continuously active, causing the observed signal to become a complex composite that is difficult to estimate. Furthermore, as fMRI data processing typically requires extensive computation to derive statistical patterns, preselecting an incorrect model order can compromise the validity of the research findings. Consequently, determining a suitable model order a priori represents a significant challenge and a key issue in current research when dealing with high-dimensional, complex signals. Presently, the primary approach for estimating model order relies on algorithms based on information-theoretic criteria (ITC). Common ITC approaches include the Akaike Information Criterion (AIC) [102], Bayesian Information Criterion (BIC) [103], and Minimum Description Length (MDL) [104]. Although they share similar mathematical forms, these criteria rely on different assumptions and support different analysis goals. AIC, derived from information theory, seeks to minimize expected information loss and applies a relatively weak penalty on model complexity, often favoring higher-dimensional models and performing well when prediction is the primary objective or when sample sizes are large. In contrast, BIC incorporates a stronger complexity penalty that increases with the logarithm of sample size and is rooted in Bayesian principles, making it more conservative and better suited for situations where avoiding overfitting is critical or where the true model is assumed to be among the candidates. MDL, closely related to BIC but motivated by data compression theory, selects the model yielding the shortest overall code length for jointly describing the model and data, thereby favoring more parsimonious representations and performing well when redundancy and noise reduction are desired. Understanding these differences allows researchers to choose an appropriate criterion based on whether the priority is predictive performance, robustness against overfitting, or parsimonious component estimation.

Beyond information-theoretic criteria, different decomposition families adopt method-specific strategies for selecting model order, each tailored to their underlying assumptions and generative frameworks. For ICA and PICA, widely used quantitative criteria include MDL, BIC, and Laplace approximation, which estimate the dimensionality that best balances model complexity against data likelihood. For PCA, scree plots, explained-variance ratios, and cross-validated reconstruction error are commonly used to determine the number of meaningful components. NMF typically relies on reconstruction residuals, sparsity-based cost functions, and stability indices that assess the reproducibility of solutions across multiple initializations. Tensor decompositions such as CPD and TCA introduce additional diagnostics, including the core-consistency index, explained tensor variance, and split-half reproducibility, which help identify the rank that preserves multilinear structure without over-factoring noise. For probabilistic models such as PROFUMO, the model order is selected using evidence-based criteria such as marginal likelihood or evidence lower bound (ELBO) convergence, which naturally penalize overly complex models. Together, these complementary strategies provide practical and method-specific guidance for determining dimensionality in real fMRI applications, addressing the challenges posed by high-dimensional and heterogeneous brain signals.

### Reproducibility

Reproducibility refers to the ability of an algorithm to yield consistent results under different time points or experimental conditions. This stability is a crucial foundation for the credibility and reliability of scientific findings. It is important to distinguish reproducibility from repeatability, as the two are closely related yet distinct. Repeatability emphasizes the consistency of results under identical experimental conditions, whereas reproducibility focuses on whether independent research teams can obtain similar results in different environments.

Numerous studies have indicated that reproducibility has become a major challenge in neuroimaging research [105, 106]. This issue is particularly pronounced in functional brain imaging. Neuroimaging data are typically high-dimensional, and many current analytical methods exhibit relatively low statistical power [107]. The high flexibility in data analysis pipelines, combined with the potential for introducing artificial artifacts during processing, contributes to low reproducibility in neuroimaging studies [108].

To address this challenge, researchers are exploring various strategies to enhance the stability and reproducibility of analytical algorithms. These strategies include employing higher-precision numerical computation methods to reduce errors, refining algorithm initialization processes to decrease dependence on specific initial values, and developing more sophisticated algorithmic structures to mitigate information loss that may arise from model simplifications. Furthermore, adopting more transparent data processing and analysis pipelines is also regarded as a key factor in improving reproducibility. Such measures can facilitate more effective validation of complex neuroscientific theories and strengthen the validity of the associated algorithms. Multiple approaches exist for evaluating reproducibility, including the use of cross-validation [109], test–retest designs [110, 111], and hierarchical clustering [100] to assess the reproducibility of single-dimensional outcomes. For simultaneously evaluating the reproducibility of multi-dimensional results, tensor clustering algorithms based on correlation or tensor spectral clustering (TSC) [14] can be employed.

Ensuring reproducibility is a critical concern in fMRI decomposition studies, given the high dimensionality and complexity of the data. To enhance reproducibility, several practical strategies can be employed. First, standardized preprocessing pipelines should be adopted, including consistent motion correction, spatial normalization, temporal filtering, and artifact removal, so that results are not confounded by differences in data preparation. Second, sharing open-source implementations of decomposition algorithms and analysis scripts enables other researchers to replicate findings directly, fostering transparency and methodological rigor. Third, the use of benchmark datasets allows performance comparisons across methods and laboratories, providing a reference point for evaluating robustness and generalizability. By integrating these practices into decomposition studies, researchers can improve both the reliability of extracted brain states and the interpretability of cross-study comparisons.

### Consistency between data decomposition and clustering

Data decomposition algorithms transform data into alternative representations, typically aiming to reduce dimensionality, extract meaningful features, or separate signal components. Clustering, an unsupervised learning method, involves partitioning a dataset into distinct groups or clusters based on specific criteria (e.g., distance) to maximize intra-cluster similarity and inter-cluster differences. Common clustering algorithms include K-means [112], hierarchical clustering [113], and DENsity-based CLUstEring (DENCLUE) [114].

Although clustering and data decomposition methods differ substantially in their underlying principles and computational procedures, they share a conceptual similarity in terms of their final outputs. Clustering algorithms typically operate by iteratively minimizing a distance-based objective function to assign data points to groups, whereas decomposition techniques such as ICA or matrix factorization aim to represent data as a combination of latent components with specific statistical properties (e.g., independence or sparsity). Both clustering and decomposition approaches ultimately partition or represent the data into meaningful units. Recognizing this high-level correspondence is useful in the context of brain-state extraction, where clustering methods have been widely employed. By acknowledging the complementary strengths of the two classes of methods, researchers may consider leveraging decomposition-based techniques to address certain limitations inherent in clustering and thereby achieve more robust and informative brain-state representations.

Similar to clustering, data decomposition methods break down the original data into multiple distinct components through a series of transformations. These components are mutually non-interfering and linearly independent. Clustering algorithms also follow this principle, whereby the original observation matrix is partitioned into independent classes via transformation matrices. However, unlike in data decomposition, the coefficient matrix in clustering is typically binary, representing the membership relationship between samples and clusters. In data decomposition, coefficient is a weight matrix, making the reconstructed data a weighted combination of the components. Consequently, by appropriately defining the objective function, a data decomposition algorithm can be adapted to achieve a clustering effect. Thus, although data decomposition and clustering differ in their primary goals and applications, they often employ similar mathematical transformations or algorithmic frameworks for data processing, which enables both to effectively uncover hidden structures and relationships within the data.

### Consistency between data decomposition and regression

Regression is a machine learning method used to model the relationship between input features and a continuous output variable. In other words, a regression model aims to characterize the underlying relationship between independent variables (predictors) and a dependent variable (outcome). Regression algorithms can be applied to tasks such as prediction, classification, and association analysis. Common regression techniques include linear regression, logistic regression, and support vector regression. In fMRI data analysis, regression algorithms are employed to model and predict relationships between brain activity and behavioral, cognitive, or stimulus-related variables.

In fMRI studies, detecting brain activation typically involves searching for signal changes associated with specific events [115]. Friston et al. (1994) proposed the General Linear Model (GLM) to estimate the interaction between the BOLD signal and experimental events by using regressors derived from events convolved with a hemodynamic response function (HRF) [116]. However, when the actual hemodynamic response shape deviates substantially from the assumed shape [117, 118], this interaction may not be effectively detected. The GLM can be considered a generalized form of regression analysis. Indeed, the standard linear regression model constitutes a special case of the GLM. This broader framework accommodates error models with non-normal distributions for the response variable and relates the mean of the distribution to the predictor variables via a link function, thereby extending the scope and applicability of regression analysis to diverse data types.

Although data decomposition and regression algorithms differ in their objectives and methodologies when processing fMRI data, they can be described within a shared model framework. The primary distinction lies in whether the parameter matrix is estimated from the data or provided as prior information. Linear regression, for instance, estimates functional connectivity between voxels by calculating correlations among their time series. If the time series of one voxel can be linearly predicted by those of others, a functional correlation between them is suggested. This approach can also be used to identify and delineate functionally connected regions. In contrast, data decomposition algorithms reveal distinct functional networks or activity patterns by breaking down complex fMRI data into multiple brain states. Whether employing regression or decomposition algorithms, the core of the analysis involves examining fMRI time series to uncover underlying brain functional networks. Both approaches must handle the complexity of high-dimensional data and aim to extract meaningful components or relationships from it. Furthermore, they rely on linear algebra and matrix operations for model fitting and evaluation, processing and analyzing the data to reveal significant patterns and structures within fMRI datasets.

### Consistency between data decomposition and fourier transform

The Fourier transform is a method for converting a signal from the time domain to the frequency domain by representing it as a sum of sinusoidal functions with different frequencies. This transform reveals the spectral information of the signal, enabling analysis of its frequency components and spectral characteristics. Fourier transforms have a broad range of applications across numerous fields, including signal processing, image processing, communication systems, and quantum mechanics. In fMRI, the Fourier transform is used to convert time-domain data into frequency-domain representations, a key step in image reconstruction. Simultaneously, complex-valued measurement data in MRI are transformed into spatial-domain images via the inverse Fourier transform, thereby generating detailed images of internal anatomical structures [119].

Although data decomposition methods and the Fourier transform differ in their objectives and underlying principles when applied to fMRI data, both involve converting signals from one representational form to another to facilitate more effective analysis and interpretation. The Fourier transform reveals the frequency components and periodic characteristics of fMRI signals by converting them from the time domain to the frequency domain, thereby enabling the identification of oscillatory patterns in brain activity. In contrast, data decomposition methods uncover distinct functional networks or activity patterns by breaking down complex fMRI data into multiple independent components, which allows for the separation of independent source signals from the observed mixture. Furthermore, both approaches seek to enhance the understanding of signals by altering their representation. They rely on linear algebra and matrix operations to process high-dimensional data through mathematical transformations and to extract meaningful components or features. Consequently, each contributes to improving the accuracy and reliability of fMRI signal analysis, albeit through different mechanistic pathways.

Beyond their apparent methodological differences, data decomposition, clustering, regression, and Fourier analysis exhibit a high degree of conceptual consistency at an abstract level, as they can all be viewed as different ways of projecting high-dimensional fMRI data into lower-dimensional representations of brain states. Clustering emphasizes discrete state partitioning, regression focuses on explaining variance with respect to external variables, and Fourier-based analysis characterizes brain dynamics in the frequency domain. Notably, these three approaches have been extensively adopted in existing fMRI-based brain state studies, each offering distinct advantages. Data decomposition provides a particularly flexible and unifying framework within which these complementary strengths can be integrated. For example, regression variables can be explicitly incorporated as constraints or priors during decomposition, frequency-domain representations can be introduced by mapping the data into Fourier space before or during factorization, and the resulting components can be transformed using SoftMax or related constraints to induce discrete or quasi-discrete state representations, effectively achieving a clustering-like effect. By embedding regression, Fourier-domain representations, and clustering-inspired constraints into the decomposition process itself, future brain state analysis methods can jointly leverage the strengths of all four paradigms. Such integrated decomposition frameworks hold the potential to more precisely extract task-relevant or clinically meaningful brain states, while maintaining interpretability and flexibility across different experimental designs.

## Conclusion and future work

This review has systematically summarized nine representative data decomposition methods for estimating brain states from fMRI data, including EMD, ICA, PCA, PROFUMO, NMF, Dictionary Learning, Tensor ICA, TCA, and SNTD. These techniques provide a data-driven means of uncovering latent spatiotemporal structures in neural signals. Compared to widely used methods such as clustering, regression-based approaches, or Fourier transforms, data decomposition methods exhibit a high degree of conceptual consistency in their shared objective of reducing the dimensionality of complex brain activity and extracting interpretable patterns. However, unlike clustering or regression, decomposition methods are particularly well-suited for capturing overlapping, temporally continuous, and spatially distributed components of brain states, offering richer and more nuanced representations of brain dynamics. Despite these advantages, challenges remain. Issues such as model order selection and the reproducibility of estimated brain states represent critical limitations that affect the reliability and interpretability of results. While matrix-based decomposition methods have been extensively validated, many tensor decomposition models—such as Tucker decomposition, tensor ring, tensor train, and joint tensor analysis—remain underexplored for brain state estimation. Future research should aim to better integrate these advanced tensor models into fMRI analysis pipelines. Furthermore, the manner in which fMRI data are structured into tensors significantly influences the outcome of decomposition. Consequently, the design and optimization of tensor construction strategies constitute an important direction for improving the interpretability and effectiveness of brain state estimation from high-dimensional neuroimaging data.

Looking forward, the field of brain state estimation is likely to benefit from integrating traditional decomposition methods with emerging computational approaches. Deep matrix and tensor factorization techniques can capture complex, hierarchical patterns in high-dimensional fMRI data, extending beyond the capabilities of linear decompositions. Variational autoencoders (VAEs) offer flexible latent representations while modeling uncertainty, enabling more robust and interpretable source separation. Additionally, foundation models trained on large-scale neuroimaging datasets provide transferable representations that can facilitate cross-study generalization and multimodal integration. By combining these modern approaches with classical methods such as PCA, ICA, TCA, and NMF, future studies may leverage their complementary strengths, improving the precision, scalability, and interpretability of brain state analysis. Such integration also opens avenues for linking decomposition results with predictive modeling, clustering, and regression frameworks, further enhancing the utility of decomposition-based analyses in neuroscience.

## Supplementary Information

Supplementary Material 1.

## Data Availability

No datasets were generated or analysed during the current study.

## Appendix

In this appendix, we provide detailed mathematical formulations for all signal decomposition methods considered in this study. For each method, the core optimization objectives and modeling assumptions are presented using a unified notation where possible, thereby facilitating direct comparison across different decomposition frameworks.

## One-dimensional approaches

### Empirical mode decomposition

For a real-valued BOLD time series, $x\left(t\right)$, the standard EMD will find a set of *R* IMFs $\{{IMF}_{i}(t)\}$, $i=1$ to $K$, which represent specific frequency bands, and a monotonic residual signal $r(t)$, so that:

1 $$x\left(t\right)={\sum }_{i=1}^{R}{IMF}_{i}\left(t\right)+r(t)$$

To ensure that the time spectrum produces meaningful frequency estimates,$\{{IMF}_{i}(t)\}$ are defined to have symmetrical upper and lower envelopes. To extract IMFs using EMD, an iterative method called the screening algorithm is used. The specific screening process is as follows: First, for a given time series$x(t)$, find the maximum and minimum points of the signal. Then, interpolate between all minimum (or maximum) values to obtain the maximum envelope ${e}_{max}(t)$ and the minimum envelope${e}_{min}(t)$. Next, average the two envelopes to obtain the average line${e}_{mean}\left(t\right)=({e}_{min}\left(t\right)+{e}_{max}\left(t\right))/2$. Subtract the average line from the original signal to obtain a new function$r(t) = x(t) - {e}_{mean}\left(t\right)$. If $r(t)$ meets the stopping criteria, it is considered to be an ${IMF}_{i}(t)$, otherwise $x(t)$ is replaced by $r(t)$ and the above process is repeated. To obtain the remaining IMFs, the same procedure is applied to the residual $r(t) = x(t) - {IMF}_{i}(t)$ until all IMFs are extracted. Further details of the EMD algorithm can be found at http://themis.ss.ncu.edu.tw/e_idl_gui_for_hht.html.

### Two-dimensional approaches

Assuming that the observed signal is a linear mixture of several source signals, the observed data can be expressed as a linear combination of these source signals through an unknown mixing matrix:

2 $$\mathbf{X}=\mathbf{A}\mathbf{S}$$

where the observation data matrix, ${\mathbf{X}\in {\mathbb{R}}}^{ M\times N}$, fMRI data reorganized into a $M$ time points by $N$ voxels data matrix. The source signal matrix $\mathbf{S}\in {\mathbb{R}}^{ R\times N}$ represents the potential brain functional network or activity patterns, with one spatial map in each row. The mixing matrix, ${\mathbf{A}\in {\mathbb{R}}}^{ M\times \mathrm{R}}$, describes the temporal dynamics of each of the spatial maps, Here, $R$, equal to the number of components (also referred to as the model order), is a parameter that must be predefined by the user. Through the observation data $\mathbf{X}$, the demixing matrix ${\mathbf{W}\in {\mathbb{R}}}^{ R\times M}$, which is estimated to recover the source components, to obtain the source components $\mathbf{S}$:

3 $$\mathbf{S}=\mathbf{W}\mathbf{X}$$

Based on the general two-dimensional formulation described above, the various decomposition methods considered in this study can be viewed as specific instantiations of this unified model.

### Independent component analysis

The basic principle behind ICA is to transform the observed data into components that are as independent as possible through linear transformation. The ICA model adopted in Eq. (2) follows the standard noiseless formulation and does not explicitly include a noise model, which may lead to overfitting and does not readily support statistical evaluation of the resulting components, such as distinguishing neural signals from background noise in spatial maps. Although ICA can in principle be extended to incorporate noise with a general covariance structure, such extensions are not explicitly considered in the formulation used here. Probabilistic ICA provides a practical and widely used alternative by introducing an explicit probabilistic generative model with a noise term, thereby improving robustness to overfitting and enabling principled statistical inference of the signal sources represented in each spatial map. The software tool for applying noise-free ICA to fMRI data is available at https://trendscenter.org/software/gift and for applying PICA at https://fsl.fmrib.ox.ac.uk/fsl/fslwiki/MELODIC.

### Principal component analysis

In the traditional PCA algorithm, the original fMRI data $\mathbf{X}$ are projected into a new coordinate system through a linear transformation. The axes of this new coordinate system are determined by the directions of maximum variance in the data, namely the principal components. The fMRI data can be centered so that the mean of the activation value of each voxel is zero. The specific formula is:

4 $${{\boldsymbol{X}}}_{centered}=\mathbf{X}-1{{\boldsymbol{\upmu}}}^{\mathrm{T}}$$

where ${{\boldsymbol{\upmu}}}^{\mathrm{T}}$ is a $1\times N$ vector, representing the mean vector of each column, and $1$ is an $M\times 1$ vector of all ones. Then calculate the covariance matrix $\mathbf{C}$ of the centered data matrix ${\mathbf{X}}_{centered}$:

5 $$\mathbf{C}=\frac{1}{N-1}{\mathbf{X}}_{centered}{\mathbf{X}}_{centered}^{\mathrm{T}}$$

Perform singular value decomposition on the covariance matrix $\mathbf{C}{\in {\mathbb{R}}}^{ M\times M}$ to obtain eigenvalues and eigenvectors:

6 $$\mathbf{C}=\mathbf{V}\mathbf{D}{\mathbf{V}}^{\mathrm{T}}$$

where ${\mathbf{V}\in {\mathbb{R}}}^{ M\times M}$ is the eigenvector matrix, $\mathbf{D}{\in {\mathbb{R}}}^{ M\times M}$ is a diagonal matrix, and the elements on the diagonal are eigenvalues. According to the size of the eigenvalue, the first $R$ principal components are selected, and the corresponding eigenvectors are ${\mathbf{V}}_{R}$∈${\mathbb{R}}^{ R\times M}$, which constitute the principal components of the data. Project the original data $\mathbf{X}$ onto the selected principal components to obtain the reduced-dimensional data matrix $\mathbf{S}{\in {\mathbb{R}}}^{ R\times N}$:

7 $$\mathbf{S}={\mathbf{V}}_{R}\mathbf{X}$$

where ${\mathbf{V}}_{R}$ is an orthogonal matrix composed of the selected $R$ principal components, which corresponding to $\mathbf{W}$ in Eq. (3). Through the above steps, PCA can extract a low-dimensional representation reflecting the main change pattern from high-dimensional fMRI data. A MATLAB toolbox containing the PCA method is available at https://www.mathworks.com/matlabcentral/fileexchange/134751-pca-toolbox-for-matlab.

### Probabilistic functional modes

The core idea of PROFUMO is to infer functional patterns by explicitly modeling the probabilistic generative process of subject-level fMRI data. Specifically, PROFUMO assumes that the fMRI data of each subject can be expressed as a linear combination of multiple latent functional patterns. For each subject $q$, the observed data ${\mathbf{X}}_{q}$ are modeled as the product of subject-specific spatial maps and corresponding temporal time courses. Within this Bayesian framework, PROFUMO jointly infers subject-specific spatial representations ${\mathbf{A}}_{q}$ and group-level functional patterns $\mathbf{S}$ that are shared across subjects, enabling the characterization of both inter-subject variability and common functional structure. Specifically, PROFUMO assumes that functional patterns have prior distributions and uses observed data to update these prior distributions. Assume that the prior distributions of temporal patterns and spatial patterns are:

8 $$\mathrm{p}\left({\mathbf{A}}_{q}\right)=\mathcal{N}\left({\mathbf{A}}_{q}\right|0,{\mathbf{C}}_{\mathbf{A}})$$

9 $$\mathrm{p}\left(\mathbf{S}\right)=\mathcal{N}\left(\mathbf{S}\right|0,{\mathbf{C}}_{\mathbf{S}})$$

where $\mathcal{N}$ represents a normal distribution. ${\mathbf{C}}_{\mathbf{A}}$ and ${\mathbf{C}}_{\mathbf{S}}$ are the covariance matrices of the temporal and spatial patterns, respectively. Third, the posterior distribution is inferred. Given the observed data ${\mathbf{X}}_{q}$, PROFUMO calculates the posterior distribution of the temporal and spatial patterns through Bayesian inference:

10 $$\mathrm{p}\left({\mathbf{A}}_{q},\mathbf{S}|{\mathbf{X}}_{q}\right)\propto \mathrm{p}\left({\mathbf{X}}_{q}|{\mathbf{A}}_{q},\mathbf{S}\right)\mathrm{p}\left({\mathbf{A}}_{q}\right)\mathrm{p}\left(\mathbf{S}\right)$$

where $\mathrm{p}\left({\mathbf{X}}_{q}|{\mathbf{A}}_{q},\mathbf{S}\right)$ is the likelihood function, which represents the probability of observing data given the temporal pattern and spatial pattern. $\mathrm{p}({\mathbf{A}}_{q})$ and $\mathrm{p}(\mathbf{S})$ are prior distributions. Through these steps, PROFUMO models the fMRI data of the subject as a linear combination of multiple functional patterns and uses Bayesian inference to infer the probability distribution of these patterns. This can better understand the functional connections and network structures between brain regions. The analysis code for the PROFUMO method is available at https://git.fmrib.ox.ac.uk/samh/profumo.

### Nonnegative matrix factorization

A key distinction between non-negative matrix factorization (NMF) and other decomposition methods lies in its explicit non-negativity constraints on both the basis and coefficient matrices. In practice, these constraints are typically satisfied by applying standard preprocessing and, when necessary, time–frequency transformations to the data, thereby ensuring that the input matrix contains only non-negative values prior to factorization. The time–frequency data matrix ${\mathbf{X}}_{TF}$ is decomposed into the product of two non-negative matrices by NMF:

11 $${\mathbf{X}}_{TF}\approx \mathbf{W}\mathbf{H}$$

where $\mathbf{W}$∈${\mathbb{R}}^{ M\times R}$ is the basis matrix, which represents the weight of $R$ basis functions in time, $\mathbf{H}$∈${\mathbb{R}}^{ R\times N}$ is the coefficient matrix, which represents the distribution of each basis function in space. Here $R$ is the number of selected basis functions. For the iterative formula of NMF, the matrix decomposition problem can be converted into the problem of minimizing the error between two matrices, that is:

12 $$\underset{\mathbf{W},\mathbf{H}}{\mathrm{min}}{\Vert {\mathbf{X}}_{TF}-\mathbf{W}\mathbf{H}\Vert }_{F}^{2}$$

During the optimization process, all elements in $\mathbf{W}$ and $\mathbf{H}$ are required to be non-negative. The iterative formulas of matrix $\mathbf{W}$ and matrix $\mathbf{H}$ are:

13 $${{\mathbf{H}}_{ij}^{k+1}={\mathbf{H}}_{ij}^{k}\frac{{({({\mathbf{W}}^{k})}^{\mathrm{T}}\mathbf{X})}_{ij}}{{({({\mathbf{W}}^{k})}^{\mathrm{T}}{\mathbf{W}}^{k}{\mathbf{H}}^{k})}_{ij}}\mathbf{W}}_{ij}^{k+1}={\mathbf{W}}_{ij}^{k}\frac{{(\mathbf{X}{({\mathbf{H}}^{k})}^{\mathrm{T}})}_{ij}}{{({{\mathbf{W}}^{k}{\mathbf{H}}^{k}({\mathbf{H}}^{k})}^{\mathrm{T}})}_{ij}}$$

where $k$ represents the number of iterations, ${\mathbf{H}}_{ij}$ represents the element in the *i*^th^ row and *j*^th^ column of the matrix $\mathbf{H}$, and ${\mathbf{W}}_{ij}$ represents the element in the *i*^th^ row and *j*^th^ column of the matrix $\mathbf{W}$. A MATLAB toolbox containing various NMF techniques and various NMF-based data mining methods is available at https://sites.google.com/site/nmftool.

### Dictionary learning

Semi-ODL combines semi-supervised learning and online dictionary learning, and is designed to handle scenarios with partially labeled data. Its core goal is to learn an effective dictionary from the data through sparse coding, and optimize the dictionary and encoding process through gradual updates. In semi-blind online dictionary learning, we usually need to minimize the following objective function:

14 $$\underset{\mathbf{D},\mathbf{X}}{\mathrm{min}}\frac{1}{2}{\Vert \mathbf{X}-\mathbf{A}\mathbf{S}\Vert }_{\mathrm{F}}^{2}+\lambda {\Vert \mathbf{S}\Vert }_{1}$$

where $\mathbf{A}$ denotes the learned dictionary and $\mathbf{S}$ is the sparse coding matrix. $\lambda$ is the sparsity regularization parameter. In each iteration, sparse coding is first performed, and its goal is to update the coding matrix $\mathbf{S}$ by solving the following problem:

15 $${\mathbf{S}}^{\mathbf{*}}=\underset{\mathbf{A}}{\mathrm{argmin}}\frac{1}{2}{\Vert \mathbf{X}-\mathbf{A}\mathbf{S}\Vert }_{\mathrm{F}}^{2}+\lambda {\Vert \mathbf{S}\Vert }_{1}$$

The dictionary is updated based on the current sparse coding matrix $\mathbf{S}$. The update formula of the dictionary matrix $\mathbf{A}$ can be expressed as:

16 $${\mathbf{A}}^{\mathbf{*}}=\underset{\mathbf{A}}{\mathrm{argmin}}\frac{1}{2}{\Vert \mathbf{X}-\mathbf{A}\mathbf{S}\Vert }_{\mathrm{F}}^{2}$$

The dictionary learning algorithm can be downloaded from http://spams-devel.gforge.inria.fr.

### Clustering

The basic idea of clustering algorithms is to divide data into K clusters so that the distance within each cluster is minimized or the similarity is maximized. The definitions of distance and similarity vary, which directly affects the fundamental goal of clustering. Same as data decomposition, clustering can also be represented with Eq. (3), where $\mathbf{S}$ is the centroid of the cluster, which represents the center point of each cluster, usually the mean of the samples belonging to that class. $\mathbf{X}$ is the sample to be clustered. $\mathbf{W}$ is the index used to identify the cluster to which each sample belongs.

17 $$\mathbf{W}=\left[\begin{array}{ccc}0/1& 0/1& \begin{array}{cc}\ddots & 0/1 \end{array}\\ 0/1& 0/1& \begin{array}{cc} \ddots & 0/1\end{array}\\ \begin{array}{c}\vdots \\ \vdots \end{array}& \begin{array}{c}\vdots \\ \vdots \end{array}& \begin{array}{cc}\begin{array}{c}\ddots \\ \ddots \end{array}& \begin{array}{c}\vdots \\ 0/1\end{array}\end{array}\end{array}\right]$$

### Regression

In standard linear regression, the goal is to find a set of weights such that the linear combination of these weights and the input variables (features) is as close as possible to the output variable (target value). From a matrix perspective, linear regression can also be formalized as a matrix decomposition as in Eq. (3) where $\mathbf{S}$ is the vector (or matrix) of the target variable or response variable, which represents the actual observed value of the model. $\mathbf{W}$ is the design matrix given by the experiment. Solve the linear regression in the sense of least squares to calculate $\mathbf{S}$. Compared with the regression algorithm, the data decomposition method estimates $\mathbf{W}$ by optimizing by setting different constraints to obtain the corresponding $\mathbf{S}$, so the two are highly consistent in principle.

### Fourier transform

The conversion from time domain to frequency domain in Fourier transform can be expressed as:

18 $$X\left(k\right)=\sum_{n=0}^{{N}_{F}-1} x(n){e}^{\mathrm{-j}\left(2\pi /{N}_{F}\right)nk}$$

where $X\left(k\right)$ is the signal representation in the frequency domain, and $x(n)$ is the time series. ${e}^{\mathrm{-j}\left(2\pi /{N}_{F}\right)nk}$ is a complex exponential function, which represents the complex form of the frequency component, $j$ is the imaginary unit, ${N}_{F}$ is the number of Fourier transform points. Similar to data decomposition, Fourier transform can be represented by the unified formula as in Eq. (3). In this case, $\mathbf{X}$ represents the time domain signal in each column and $\mathbf{S}$ represents the frequency domain signal. The function $\mathbf{W}$ converts the time domain signal into the frequency domain signal, describing the composition of the signal in the frequency domain:

19 $${\mathbf{W}} = \left[ {\begin{array}{*{20}c} {e^{{ - i(\frac{2\pi }{N}*0*0)}} } & {e^{{ - i(\frac{2\pi }{N}*0*1)}} } & {\begin{array}{*{20}c} \ddots & {e^{{ - i(\frac{2\pi }{N}*0*(n - 1))}} } \\ \end{array} } \\ {e^{{ - i(\frac{2\pi }{N}*1*0)}} } & {e^{{ - i(\frac{2\pi }{N}*1*1)}} } & {\begin{array}{*{20}c} \ddots & {e^{{ - i(\frac{2\pi }{N}*1*(n - 1))}} } \\ \end{array} } \\ {\begin{array}{*{20}c} \vdots \\ \vdots \\ \end{array} } & {\begin{array}{*{20}c} \vdots \\ \vdots \\ \end{array} } & {\begin{array}{*{20}c} {\begin{array}{*{20}c} \ddots \\ \ddots \\ \end{array} } & {\begin{array}{*{20}c} \vdots \\ {e^{{ - i(\frac{2\pi }{N}*(n - 1)*(n - 1))}} } \\ \end{array} } \\ \end{array} } \\ \end{array} } \right]$$

Therefore, Fourier transform can be viewed as a special case of data decomposition, which can be used to map the signal to the frequency space. With data decomposition, the signal can be mapped to any space.

## Multi-dimensional apporaches

For a third-order fMRI tensor $\underset{\_}{\mathbf{X}}$ (such as the three dimensions of time, space, and subject), the basic CPD formula can be expressed as:

20 $$\underset{\_}{\mathbf{X}}=\underset{\_}{\mathbf{I}}{\times }_{1}\mathbf{A}{\times }_{2}\mathbf{B}{\times }_{3}\mathbf{C}+\underset{\_}{\mathbf{E}}={\sum }_{r=1}^{R}{{\boldsymbol{a}}}_{r}\circ {{\boldsymbol{b}}}_{r}\circ {{\boldsymbol{c}}}_{r}+\underset{\_}{\mathbf{E}}$$

where $\mathbf{A}$∈${\mathbb{R}}^{I \times R}, \mathbf{B}$∈${\mathbb{R}}^{J \times R}, \mathbf{C}$∈${\mathbb{R}}^{K \times R}$. $\underset{\_}{\mathbf{X}}$∈${\mathbb{R}}^{I \times J \times K}$ is the original data tensor, containing $I$ spatial nodes, $J$ time points and $K$ subjects and${{\boldsymbol{a}}}_{r}\in {\mathbb{R}}^{I\times 1 }, {{\boldsymbol{b}}}_{r}\in {\mathbb{R}}^{J\times 1 }, {{\boldsymbol{c}}}_{r}\in {\mathbb{R}}^{K\times 1}$. $\underset{\_}{\mathbf{E}}$∈${\mathbb{R}}^{I \times J \times K}$ is the residual tensor, which represents the noise or error that the model cannot explain. The symbol $\circ$ represents the outer product and ${\times }_{i}$ represents the product of a tensor's *i*^th^ dimension with a matrix. Based on the general multi-dimensional formulation described above, tensor ICA, TCA, SNTD can be viewed as specific instantiations of this unified model.

### Tensor independent component analysis

Tensor ICA works by decomposing the data tensor into source tensors and mixing matrices. The key to Tensor ICA is that the independent components in the source tensor are as statistically independent as possible. In tensor ICA, the independent constrain is applied in spatial mode${{\boldsymbol{a}}}_{r}$. The installation package of the Tensor ICA algorithm is available at https://aeteschendorff-lab.github.io/software/tensorICA.

### Tensor component analysis

Alternating least squares (ALS) is an optimization method commonly used in TCA. The objective function of TCA is usually:

21 $$\underset{\mathbf{A},\mathbf{B},\mathbf{C}}{\mathrm{min}}\frac{1}{2}{\Vert \underset{\_}{\mathbf{X}}-{\sum }_{r=1}^{R}{{\boldsymbol{a}}}_{r}\circ {{\boldsymbol{b}}}_{r}\circ {{\boldsymbol{c}}}_{r}\Vert }_{\mathrm{F}}^{2}$$

The analysis code for the TCA framework is available at https://github.com/GHu-DUT/Tensor-components-analysis-for-naturalistic-stimuli-fMRI.

### Sparse nonnegative tensor decomposition

The implementation of the SNTD method consists of constructing a spatiotemporal frequency data tensor that aggregates all subjects, and then applying SNTD to extract frequency-specific co-activation patterns (CAP). Specifically, the SNTD method includes the following Steps: First, construct a tensor from the preprocessed fMRI data, convert the time series of each subject's brain nodes into the time–frequency domain, and then stack the data of all nodes to construct a three-dimensional tensor ${\underset{\_}{\mathbf{X}}}_{TF}\in {\mathbb{R}}^{S\times F\times N}$ which contains $S$ spatial nodes, $F$ frequency bins and $N$ time points. Then subject tensor can be concatenated in the temporal domain and the aggregate tensor can be represented as $\underset{\_}{\mathbf{X}}\in {\mathbb{R}}^{S\times F\times NI}$, where $I$ represents the number of subjects. The constructed tensor is then decomposed using the SNTD method:

22 $${\underset{\_}{\mathbf{X}}}_{TF}={\sum }_{r=1}^{R}{{\boldsymbol{s}}}_{r}\circ {{\boldsymbol{f}}}_{r}\circ {{\boldsymbol{t}}}_{r}$$

where ${{\boldsymbol{s}}}_{r}\in {\mathbb{R}}^{1\times S }{, {\boldsymbol{f}}}_{r}\in {\mathbb{R}}^{1\times F }{, {\boldsymbol{t}}}_{r}\in {\mathbb{R}}^{1\times NI}$ represent the spatial, frequency, temporal & subject pattern vectors of the r^th^ component respectively. The objective function is optimized by imposing non-negative constraints on all components. Additional sparsity constraints are applied to the temporal component to obtain the CAP at a specific frequency, along with the corresponding spatial, frequency, and temporal factor matrices.

23 $$\mathop {{\mathrm{min}}}\limits_{{{\mathbf{A}} \ge 0,{\mathbf{B}} \ge 0,{\mathbf{C}} \ge 0}} \left\| {{\mathbf{\underline {X} }}_{TF} - \sum {_{r = 1}^{R} s_{r}^\circ f_{r}^\circ t_{r} } } \right\|_{{\mathrm{F}}}^{2} + \lambda \sum {_{r = 1}^{R} } \left\| {c_{r} } \right\|_{1}$$

where $\lambda$ is a sparsity control parameter used to balance reconstruction error and sparsity. The analysis code implementing the SNTD analysis framework is available at https://github.com/GHu-DUT/Sparse-nonnegative-CPD-for-CAP.
